# Posttraumatic stress disorder: from diagnosis to prevention

**DOI:** 10.1186/s40779-018-0179-0

**Published:** 2018-09-28

**Authors:** Xue-Rong Miao, Qian-Bo Chen, Kai Wei, Kun-Ming Tao, Zhi-Jie Lu

**Affiliations:** Department of Anesthesiology and Intensive Care, Third Affiliated Hospital of Second Military Medical University, Shanghai, China

**Keywords:** PTSD, Stress, Cognitive impairment, Psychological interventions, Neuroendocrine

## Abstract

Posttraumatic stress disorder (PTSD) is a chronic impairment disorder that occurs after exposure to traumatic events. This disorder can result in a disturbance to individual and family functioning, causing significant medical, financial, and social problems. This study is a selective review of literature aiming to provide a general outlook of the current understanding of PTSD. There are several diagnostic guidelines for PTSD, with the most recent editions of the DSM-5 and ICD-11 being best accepted. Generally, PTSD is diagnosed according to several clusters of symptoms occurring after exposure to extreme stressors. Its pathogenesis is multifactorial, including the activation of the hypothalamic–pituitary–adrenal (HPA) axis, immune response, or even genetic discrepancy. The morphological alternation of subcortical brain structures may also correlate with PTSD symptoms. Prevention and treatment methods for PTSD vary from psychological interventions to pharmacological medications. Overall, the findings of pertinent studies are difficult to generalize because of heterogeneous patient groups, different traumatic events, diagnostic criteria, and study designs. Future investigations are needed to determine which guideline or inspection method is the best for early diagnosis and which strategies might prevent the development of PTSD.

## Background

Posttraumatic stress disorder (PTSD) is a recognized clinical phenomenon that often occurs as a result of exposure to severe stressors, such as combat, natural disaster, or other events [1]. The diagnosis of PTSD was first introduced in the 3rd edition of the Diagnostic and Statistical Manual (DSM) (American Psychiatric Association) in 1980 [2].

PTSD is a potentially chronic impairing disorder that is characterized by re-experience and avoidance symptoms as well as negative alternations in cognition and arousal. This disease first raised public concerns during and after the military operations of the United States in Afghanistan and Iraq, and to date, a large number of research studies report progress in this field. However, both the underlying mechanism and specific treatment for the disease remain unclear. Considering the significant medical, social and financial problems, PTSD represents both to nations and to individuals, all persons caring for patients suffering from this disease or under traumatic exposure should know about the risks of PTSD.

The aim of this review article is to present the current understanding of PTSD related to military injury to foster interdisciplinary dialog. This article is a selective review of pertinent literature retrieved by a search in PubMed, using the following keywords: “PTSD[Mesh] AND military personnel”. The search yielded 3000 publications. The ones cited here are those that, in the authors’ view, make a substantial contribution to the interdisciplinary understanding of PTSD.

## Definition and differential diagnosis

Posttraumatic stress disorder is a prevalent and typically debilitating psychiatric syndrome with a significant functional disturbance in various domains. Both the manifestation and etiology of it are complex, which has caused difficulty in defining and diagnosing the condition. The 3rd edition of the DSM introduced the diagnosis of PTSD with 17 symptoms divided into three clusters in 1980. After several decades of research, this diagnosis was refined and improved several times. In the most recent version of the DSM-5 [3], PTSD is classified into 20 symptoms within four clusters: intrusion, active avoidance, negative alterations in cognitions and mood as well as marked alterations in arousal and reactivity. The diagnosis requirement can be summarized as an exposure to a stressor that is accompanied by at least one intrusion symptom, one avoidance symptom, two negative alterations in cognitions and mood symptoms, and two arousal and reactivity turbulence symptoms, persisting for at least one month, with functional impairment. Interestingly, in the DSM-5, PTSD has been moved from the anxiety disorder group to a new category of ‘trauma- and stressor-related disorders’, which reflects the cognizance alternation of PTSD. In contrast to the DSM versions, the World Health Organization’s (WHO) International Classification of Diseases (ICD) has proposed a substantially different approach to diagnosing PTSD in the most recent ICD-11 version [4], which simplified the symptoms into six under three clusters, including constant re-experiencing of the traumatic event, avoidance of traumatic reminders and a sense of threat. The diagnosis requires at least one symptom from each cluster which persists for several weeks after exposure to extreme stressors. Both diagnostic guidelines emphasize the exposure to traumatic events and time of duration, which differentiate PTSD from some diseases with similar symptoms, including adjustment disorder, anxiety disorder, obsessive-compulsive disorder, and personality disorder. Patients with the major depressive disorder (MDD) may or may not have experienced traumatic events, but generally do not have the invasive symptoms or other typical symptoms that PTSD presents. In terms of traumatic brain injury (TBI), neurocognitive responses such as persistent disorientation and confusion are more specific symptoms. It is worth mentioning that some dissociative reactions in PTSD (e.g., flashback symptoms) should be recognized separately from the delusions, hallucinations, and other perceptual impairments that appear in psychotic disorders since they are based on actual experiences. The ICD-11 also recognizes a sibling disorder, complex PTSD (CPTSD), composed of symptoms including dysregulation, negative self-concept, and difficulties in relationships based on the diagnosis of PTSD. The core CPTSD symptom is PTSD with disturbances in self-organization (DSO).

In consideration of the practical applicability of the PTSD diagnosis, Brewin et al. conducted a study to investigate the requirement differences, prevalence, comorbidity, and validity of the DSM-5 and ICD-11 for PTSD criteria. According to their study, diagnostic standards for symptoms of re-experiencing are higher in the ICD-11 than the DSM, whereas the standards for avoidance are less strict in the ICD-11 than in the DSM-IV [5]. It seems that in adult subjects, the prevalence of PTSD using the ICD-11 is considerably lower compared to the DSM-5. Notably, evidence suggested that patients identified with the ICD-11 and DSM-5 were quite different with only partially overlapping cases; this means each diagnostic system appears to find cases that would not be diagnosed using the other. In consideration of comorbidity, research comparing these two criteria show diverse outcomes, as well as equal severity and quality of life. In terms of children, only very preliminary evidence exists suggesting no significant difference between the two. Notably, the diagnosis of young children (age ≤ 6 years) depends more on the situation in consideration of their physical and psychological development according to the DSM-5.

Despite numerous investigations and multiple revisions of the diagnostic criteria for PTSD, it remains unclear which type and what extent of stress are capable of inducing PTSD. Fear responses, especially those related to combat injury, are considered to be sufficient enough to trigger symptoms of PTSD. However, a number of other types of stressors were found to correlate with PTSD, including shame and guilt, which represent moral injury resulting from transgressions during a war in military personnel with deeply held moral and ethical beliefs. In addition, military spouses and children may be as vulnerable to moral injury as military service members [6]. A research study on Canadian Armed Forces personnel showed that exposure to moral injury during deployments is common among military personnel and represents an independent risk factor for past-year PTSD and MDD [7]. Unfortunately, it seems that pre- and post-deployment mental health education was insufficient to moderate the relationship between exposure to moral injury and adverse mental health outcomes.

In general, a large number of studies are focusing on the definition and diagnostic criteria of PTSD and provide considerable indicators for understanding and verifying the disease. However, some possible limitations or discrepancies continue to exist in current research studies. One is that although the diagnostic criteria for a thorough examination of the symptoms were explicit and accessible, the formal diagnosis of PTSD using structured clinical interviews was relatively rare. In contrast, self-rating scales, such as the Posttraumatic Diagnostic Scale (PDS) [8] and the Impact of Events Scale (IES) [9], were used frequently. It is also noteworthy that focusing on PTSD explicitly could be a limitation as well. The complexity of traumatic experiences and the responses to them urge comprehensive investigations covering all aspects of physical and psychological maladaptive changes.

## Prevalence and importance

Posttraumatic stress disorder generally results in poor individual-level outcomes, including co-occurring disorders such as depression and substance use, and physical health problems. According to the DSM-5 reporting, more than 80% of PTSD patients share one or more comorbidities; for instance, the morbidity of PTSD with concurrent mild TBI is 48% [8]. Moreover, cognitive impairment has been identified frequently in PTSD. The reported incidence rate for PTSD ranges from 5.4 to 16.8% in military service members and veterans [10–14], which is almost double those in the general population. The estimated prevalence of PTSD varies depending on the group of patients studied, the traumatic events occurred, and the measurement method used (Table 1). However, it still reflects the profound effect of this mental disease, especially with the rise in global terrorism and military conflict in recent years. While PTSD can arise at any life stage in any population, most research in recent decades has focused on returned veterans; this means most knowledge regarding PTSD has come from the military population. Meanwhile, the impact of this disease on children has received scant attention.

**Table 1:** Prevalence of PTSD in military personnel and veterans

Country	Method	Population	Sample Size (n)	Time after return	Prevalence (%)
Dutch [10]	DSM-IV	Military personnel returning from deployment to Afghanistan	994	6 months	8.9
USA [11]	DSM-IV	Military personnel returning from deployment to Iraq	1560	4 months	16.8
UK [11]	DSM-IV	Military personnel returning from deployment to Iraq	313	12 months	6.7
USA [12]	DSM-IV	Navy and Marine Corps personnel returning from deployment to Iraq, Afghanistan, or Kuwait	31,534	6 months	5.4
USA [13]	ICD-9-CM	Military personnel returning from deployment to Iraq	3403	1–6 months	16.3
USA [14]	DSM-IV	Military personnel deployed in support of the conflicts in Iraq and Afghanistan	22,630	5 years	8.1

The discrepancy of PTSD prevalence in males and females is controversial. In a large study of OEF/OIF veterans, the prevalence of PTSD in males and females was similar, although statistically more prevalent in men versus women (13% vs. 11%) [15]. Another study on the Navy and Marine Corps showed a slightly higher incidence for PTSD in the women compared to men (6.6% vs. 5.3%) [12]. However, the importance of combat exposure is unclear. Despite a lower level of combat exposure than male military personnel, females generally have considerably higher rates of military sexual trauma, which is significantly associated with the development of PTSD [16].

It is reported that 44–72% of veterans suffer high levels of stress after returning to civilian life. Many returned veterans with PTSD show emotion regulation problems, including emotion identification, expression troubles and self-control issues. Nevertheless, a meta-analytic investigation of 34 studies consistently found that the severity of PTSD symptoms was significantly associated with anger, especially in military samples [17]. Not surprisingly, high levels of PTSD and emotional regulation troubles frequently lead to poor family functioning or even domestic violence in veterans. According to some reports, parenting difficulties in veteran families were associated with three PTSD symptom clusters. Evans et al. [18] conducted a survey to evaluate the impact of PTSD symptom clusters on family functioning. According to their analysis, avoidance symptoms directly affected family functioning, whereas hyperarousal symptoms had an indirect association with family functioning. Re-experience symptoms were not found to impact family functioning. Notably, recent epidemiologic studies using data from the Veterans Health Administration (VHA) reported that veterans with PTSD were linked to suicide ideations and behaviors [19] (e.g., non-suicidal self-injury, NSSI), in which depression as well as other mood disruptions, often serve as mediating factors.

Previously, there was a controversial attitude toward the vulnerability of young children to PTSD. However, growing evidence suggests that severe and persistent trauma could result in stress responses worse than expected as well as other mental and physical sequelae in child development. The most prevalent traumatic exposures for young children above the age of 1 year were interpersonal trauma, mostly related to or derived from their caregivers, including witnessing intimate partner violence (IPV) and maltreatment [20]. Unfortunately, because of the crucial role that caregivers play in early child development, these types of traumatic events are especially harmful and have been associated with developmental maladaptation in early childhood. Maladaptation commonly represents a departure from normal development and has even been linked to more severe effects and psychopathology. In addition, the presence of psychopathology may interfere with the developmental competence of young children. Research studies have also broadened the investigation to sequelae of PTSD on family relationships. It is proposed that the children of parents with symptoms of PTSD are easily deregulated or distressed and appear to face more difficulties in their psychosocial development in later times compared to children of parents without. Meanwhile, PTSD veterans described both emotional (e.g., hurt, confusion, frustration, fear) and behavioral (e.g., withdrawal, mimicking parents’ behavior) disruption in their children [21]. Despite the increasing emphasis on the effects of PTSD on young children, only a limited number of studies examined the dominant factors that influence responses to early trauma exposures, and only a few prospective research studies have observed the internal relations between early PTSD and developmental competence. Moreover, whether exposure to both trauma types in early life is associated with more severe PTSD symptoms than exposure to one type remains an outstanding question.

## Molecular mechanism and predictive factors

The mechanisms leading to posttraumatic stress disorder have not yet been fully elucidated. Recent literature suggests that both the neuroendocrine and immune systems are involved in the formulation and development of PTSD [22, 23]. After traumatic exposures, the stress response pathways of the hypothalamic–pituitary–adrenal (HPA) axis and sympathetic nervous system are activated and lead to the abnormal release of glucocorticoids (GC) and catecholamines. GCs have downstream effects on immunosuppression, metabolism enhancement, and negative feedback inhibition of the HPA axis by binding to the GC receptor (GR), thus connecting the neuroendocrine modulation with immune disturbance and inflammatory response. A recent meta-analysis of 20 studies found increased plasma levels of proinflammatory cytokines tumor necrosis factor-alpha (TNF-a), interleukin-1beta (IL-1b), and interleukin-6 (IL-6) in individuals with PTSD compared to healthy controls [24]. In addition, some other studies speculate that there is a prospective association of C-reactive protein (CRP) and mitogen with the development of PTSD [25]. These findings suggest that neuroendocrine and inflammatory changes, rather than being a consequence of PTSD, may in fact act as a biological basis and preexisting vulnerability for developing PTSD after trauma. In addition, it is reported that elevated levels of terminally differentiated T cells and an altered Th1/Th2 balance may also predispose an individual to PTSD.

Evidence indicates that the development of PTSD is also affected by genetic factors. Research has found that genetic and epigenetic factors account for up to 70% of the individual differences in PTSD development, with PTSD heritability estimated at 30% [26]. While aiming to integrate genetic studies for PTSD and build a PTSD gene database, Zhang et al. [27] summarized the landscape and new perspective of PTSD genetic studies and increased the overall candidate genes for future investigations. Generally, the polymorphisms moderating HPA-axis reactivity and catecholamines have been extensively studied, such as FKBP5 and catechol-O-methyl-transferase (COMT). Other potential candidates for PTSD such as AKT, a critical mediator of growth factor-induced neuronal survival, were also explored. Genetic research has also made progress in other fields. For example, researchers have found that DNA methylation in multiple genes is highly correlated with PTSD development. Additional studies have found that stress exposure may even affect gene expression in offspring by epigenetic mechanisms, thus causing lasting risks. However, some existing problems in the current research of this field should be noted. In PTSD genetic studies, variations in population or gender difference, a wide range of traumatic events and diversity of diagnostic criteria all may attribute to inconsistency, thus leading to a low replication rate among similar studies. Furthermore, PTSD genes may overlap with other mental disorders such as depression, schizophrenia, and bipolar disorder. All of these factors indicate an urgent need for a large-scale genome-wide study of PTSD and its underlying epidemiologic mechanisms.

It is generally acknowledged that some mental diseases, such as major depressive disorder (MDD), bipolar disorder, and schizophrenia, are associated with massive subcortical volume change. Recently, numerous studies have examined the relationship between the morphology changes of subcortical structures and PTSD. One corrected analysis revealed that patients with PTSD show a pattern of lower white matter integrity in their brains [28]. Prior studies typically found that a reduced volume of the hippocampus, amygdala, rostral ventromedial prefrontal cortex (rvPFC), dorsal anterior cingulate cortex (dACC), and the caudate nucleus may have a relationship with PTSD patients. Logue et al. [29] conducted a large neuroimaging study of PTSD that compared eight subcortical structure volumes (nucleus accumbens, amygdala, caudate, hippocampus, pallidum, putamen, thalamus, and lateral ventricle) between PTSD patients and controls. They found that smaller hippocampi were particularly associated with PTSD, while smaller amygdalae did not show a significant correlation. Overall, rigorous and longitudinal research using new technologies, such as magnetoencephalography, functional MRI, and susceptibility-weighted imaging, are needed for further investigation and identification of morphological changes in the brain after a traumatic exposure.

## Psychological and pharmacological strategies for prevention and treatment

### Prevention

Current approaches to PTSD prevention span a variety of psychological and pharmacological categories, which can be divided into three subgroups: primary prevention (before the traumatic event, including prevention of the event itself), secondary prevention (between the traumatic event and the development of PTSD), and tertiary prevention (after the first symptoms of PTSD become apparent). The secondary and tertiary prevention of PTSD has abundant methods, including different forms of debriefing, treatments for Acute Stress Disorder (ASD) or acute PTSD, and targeted intervention strategies. Meanwhile, the process of primary prevention is still in its infancy and faces several challenges.

Based on current research on the primary prevention of post-trauma pathology, psychological and pharmacological interventions for particular groups or individuals (e.g., military personnel, firefighters, etc.) with a high risk of traumatic event exposure were applicable and acceptable for PTSD sufferers. Of the studies that reported possible psychological prevention effects, training generally included a psychoeducational component and a skills-based component relating to stress responses, anxiety reducing and relaxation techniques, coping strategies and identifying thoughts, emotion and body tension, choosing how to act, attentional control, emotion control and regulation [30–32]. However, efficiency for these training has not been evaluated yet due to a lack of high-level evidence-based studies. Pharmacological options have targeted the influence of stress on memory formation, including drugs relating to the hypothalamic-pituitary-adrenal (HPA) axis, the autonomic nerve system (especially the sympathetic nerve system), and opiates. Evidence has suggested that pharmacological prevention is most effective when started before and early after the traumatic event, and it seems that sympatholytic drugs (alpha and beta-blockers) have the highest potential for primary prevention of PTSD [33]. However, one main difficulty limiting the exploration in this field is related to rigorous and complex ethical issues, as the application of pre-medication for special populations and the study of such options in hazardous circumstances possibly touches upon questions of life and death. Significantly, those drugs may have potential side effects.

### Treatment

**Fig. 1:**
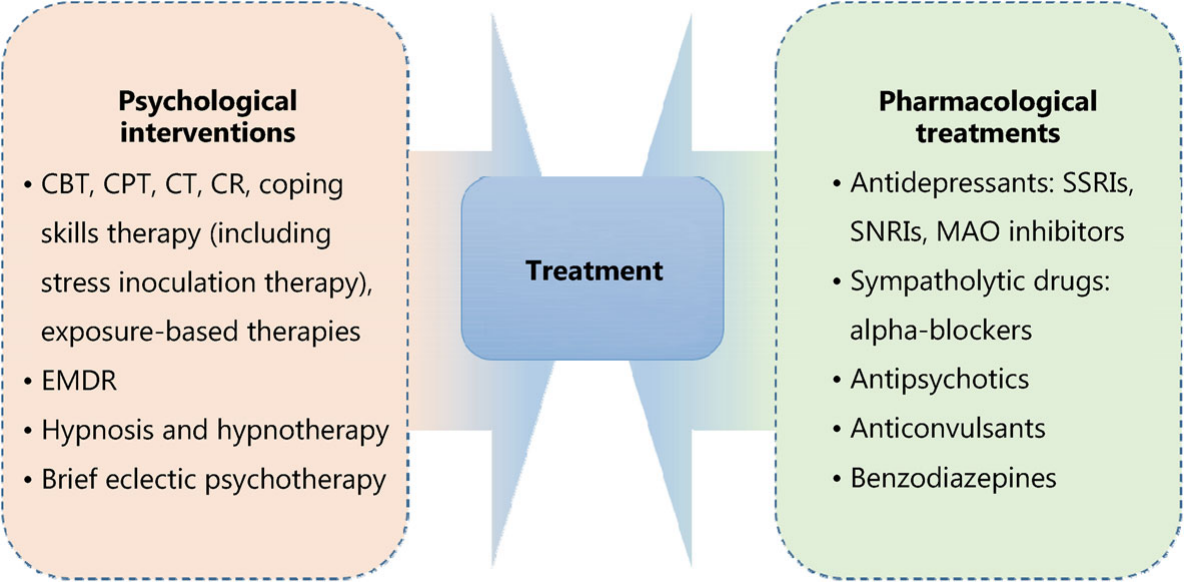
Psychological and pharmacological strategies for treatment of PTSD. CBT. Cognitive behavioral therapy; CPT. Cognitive processing therapy; CT. Cognitive therapy; CR. Cognitive restructuring; EMDR. Eye movement desensitization and reprocessing; SSRIs. Selective serotonin reuptake inhibitors; SNRIs. Serotonin and norepinephrine reuptake inhibitors; MAO. Monoamine oxidase

There are several treatment guidelines for patients with PTSD produced by different organizations, including the American Psychiatric Association (APA), the United Kingdom’s National Institute for Health and Clinical Excellence (NICE), the International Society for Traumatic Stress Studies (ISTSS), the Institute of Medicine (IOM), the Australian National Health and Medical Research Council, and the Department of Veterans Affairs and Department of Defense (VA, DoD) [34–38]. Additionally, a large number of research studies are aiming to evaluate an effective treatment method for PTSD. According to these guidelines and research, treatment approaches can be classified as psychological interventions and pharmacological treatments (Fig. 1); most of the studies provide varying degrees of improvement in individual outcomes after standard interventions, including PTSD symptom reduction or remission, loss of diagnosis, release or reduction of comorbid medical or psychiatric conditions, quality of life, disability or functional impairment, return to work or to active duty, and adverse events.

Most guidelines identify trauma-focused psychological interventions as first-line treatment options [39], including cognitive behavioral therapy (CBT), cognitive processing therapy (CPT), cognitive therapy (CT), cognitive restructuring (CR), coping skills therapy (including stress inoculation therapy), exposure-based therapies, eye movement desensitization and reprocessing (EMDR), hypnosis and hypnotherapy, and brief eclectic psychotherapy. These treatments are delivered predominantly to individuals, but some can also be conducted in family or group settings. However, the recommendation of current guidelines seems to be projected empirically as research on the comparison of outcomes of different treatments is limited. Jonas et al. [40] performed a systematic review and network meta-analysis of the evidence for treatment of PTSD. The study suggested that all psychological treatments showed efficacy for improving PTSD symptoms and achieving the loss of PTSD diagnosis in the acute phase, and exposure-based treatments exhibited the strongest evidence of efficacy with high strength of evidence (SOE). Furthermore, Kline et al. [41] conducted a meta-analysis evaluating the long-term effects of in-person psychotherapy for PTSD in 32 randomized controlled trials (RCTs) including 2935 patients with long-term follow-ups of at least 6 months. The data suggested that all studied treatments led to lasting improvements in individual outcomes, and exposure therapies demonstrated a significant therapeutic effect as well with larger effect sizes compared to other treatments.

Pharmacological treatments for PTSD include antidepressants such as selective serotonin reuptake inhibitors (SSRIs), serotonin and norepinephrine reuptake inhibitors (SNRIs), and monoamine oxidase (MAO) inhibitors, sympatholytic drugs such as alpha-blockers, antipsychotics, anticonvulsants, and benzodiazepines. Among these medications, fluoxetine, paroxetine, sertraline, topiramate, risperidone, and venlafaxine have been identified as efficacious in treatment. Moreover, in the Jonas network meta-analysis of 28 trials (4817 subjects), they found paroxetine and topiramate to be more effective for reducing PTSD symptoms than most other medications, whereas evidence was insufficient for some other medications as research was limited [40]. It is worth mentioning that in these studies, efficacy for the outcomes, unlike the studies of psychological treatments, was mostly reported as a remission in PTSD or depression symptoms; other outcomes, including loss of PTSD diagnosis, were rarely reported in studies.

As for the comparative evidence of psychological with pharmacological treatments or combinations of psychological treatments and pharmacological treatments with other treatments, evidence was insufficient to draw any firm conclusions [40]. Additionally, reports on adverse events such as mortality, suicidal behaviors, self-harmful behaviors, and withdrawal of treatment were relatively rare.

## Conclusion

PTSD is a high-profile clinical phenomenon with a complicated psychological and physical basis. The development of PTSD is associated with various factors, such as traumatic events and their severity, gender, genetic and epigenetic factors. Pertinent studies have shown that PTSD is a chronic impairing disorder harmful to individuals both psychologically and physically. It brings individual suffering, family functioning disorders, and social hazards. The definition and diagnostic criteria for PTSD remain complex and ambiguous to some extent, which may be attributed to the complicated nature of PTSD and insufficient research on it. The underlying mechanisms of PTSD involve changes in different levels of psychological and molecular modulations. Thus, research targeting the basic mechanisms of PTSD using standard clinical guidelines and controlled interference factors is needed. In terms of treatment, psychological and pharmacological interventions could relief PTSD symptoms to different degrees. However, it is necessary to develop systemic treatment as well as symptom-specific therapeutic methods. Future research could focus on predictive factors and physiological indicators to determine effective prevention methods for PTSD, thereby reducing its prevalence and preventing more individuals and families from struggling with this disorder.

## Abbreviations

APA: American Psychiatric Association
ASD: Acute stress disorder
CBT: Cognitive behavioral therapy
COMT: Catechol-O-methyl-transferase
CPT: Cognitive processing therapy
CPTSD: Complex posttraumatic stress disorder
CR: Cognitive restructuring
CRP: C-reactive protein
CT: Cognitive therapy
dACC: Dorsal anterior cingulate cortex
DSM: Diagnostic and Statistical Manual
DSO: Disturbances in self-organization
EMDR: Eye movement desensitization and reprocessing
GC: Glucocorticoids
GR: Glucocorticoids receptor
HPA-axis: Hypothalamic–pituitary–adrenal axis
ICD: International classification of diseases
IES: Impact of events scale
IL-1b: Interleukin-1beta
IL-6: Interleukin-6
IOM: Institute of Medicine
IPV: Intimate partner violence
ISTSS: International Society for Traumatic Stress Studies
MAO: Monoamine oxidase
MDD: Major depressive disorder
NICE: United Kingdom’s National Institute for Health and Clinical Excellence
NSSI: Non-suicidal self-injury
PDS: Posttraumatic diagnostic scale
PTSD: Posttraumatic stress disorder
RCTs: Randomized controlled trials
rvPFC: Rostral ventromedial prefrontal cortex
SNRIs: Serotonin and norepinephrine reuptake inhibitors;
SOE: Strength of evidence
SSRIs: Selective serotonin reuptake inhibitors
TNF-α: Tumor necrosis factor-alpha
VA: DoD Department of Veterans Affairs and Department of Defense
VHA: Veterans Health Administration
WHO: World Health Organization
